# Factors interfering with the adoption of good hygiene practices in public school food services in Bahia, Brazil

**DOI:** 10.3389/fpubh.2022.975140

**Published:** 2022-09-15

**Authors:** Jeane dos Santos Ferreira, Maria da Purificação Nazaré Araújo, Raquel Braz Assunção Botelho, Renata Puppin Zandonadi, Eduardo Yoshio Nakano, António Raposo, Heesup Han, Martín Nader, Antonio Ariza-Montes, Rita de Cássia Coelho de Almeida Akutsu

**Affiliations:** ^1^Department of Nutrition, Federal University of Sergipe, Aracaju, Brazil; ^2^School of Nutrition, Federal University of Bahia, Salvador, Brazil; ^3^Department of Nutrition, Faculty of Health Sciences, University of Brasília, Brasília, Brazil; ^4^Department of Statistics, University of Brasilia, Brasília, Brazil; ^5^CBIOS (Research Center for Biosciences and Health Technologies), Universidade Lusófona de Humanidades e Tecnologias, Lisboa, Portugal; ^6^College of Hospitality and Tourism Management, Sejong University, Seoul, South Korea; ^7^Department of Psychological Studies, Universidad ICESI, Cali, Colombia; ^8^Social Matters Research Group, Universidad Loyola Andalucía, Córdoba, Spain

**Keywords:** low-income, school food services, hygiene practices, knowledge, attitude

## Abstract

This cross-sectional study aimed to identify factors that interfere with the adoption of good hygiene practices in public school food services (SFS) in Bahia, Brazil. The search was conducted in public schools in Bahia/Brazil. Data collection included (i) evaluation of the adoption of Good Practices in school food services through visual observation and registration in the checklist in Good Hygienic Practices in School Food Services; (ii) Identification of schools' foodservice physical areas and environmental comfort measures; (iii) identification of sociodemographic and occupational characteristics and assessment of attitudes and level of knowledge in food hygiene. The minimum sample of 158 schools (confidence level of 95% and an error of 5%) was stratified considering the total number of districts (areas) and the schools' number per area. The data were analyzed using the Statistical Package for Science—SPSS® in version 26.0. The categorical variables were described as frequencies and percentages, and the quantitative variables as mean and standard deviation. Chi-square, Kruskal-Wallis with Tukey's *post-hoc* tests were used to examine qualitative variables. Independent Student *t-*test and ANOVA with Tukey's *post-hoc* tests were used to examine quantitative variables. Categorical variables were evaluated by Pearson Chi-squared tests. The Pearson test was used to analyze the correlation between Attitude and knowledge. The classification of the sanitary status was of moderate risk in 74.8% (*n* = 119) of the SFS (51-75% of compliance) and 25.2% (*n* = 40) at high risk (26-50% compliance). The average percentage of compliance for the 159 SFS in the municipality was 50.23%, obtaining a high-risk classification (26–50% compliance). In the SFS, the absence of dry goods' storage, meat preparation area, and storage of residues in more than 98% of schools was observed. Accessing attitudes, 1.4% (*n* = 2) of the food handlers were classified as unsatisfactory (0 to 49% of correct answers), 8.5% (*n* = 12) as satisfactory with restriction (50–69% of correct answers) and 90.1% (*n* = 128) as satisfactory. There was no correlation between Attitude and Knowledge level (*p* = 0.394). Considering the knowledge level, the item with the highest and lowest number of hits were: “To avoid food contamination, I wash and disinfect my hands before preparing food” and “Contaminated food will always have some change in color, smell, or taste”. There was a significant difference in the level of knowledge considering mean wage (*p* = 0.000), time working in school food service (*p* = 0.001), weekly workday (*p* = 0.000), and participation in food hygiene training (*p* = 0.000). Therefore, factors that interfered in adopting good practices in the SFS were: inadequate physical structure, absence of areas in the SFS, and absence/low number of equipment to control the production process in the cold and hot chain. Food handlers showed satisfactory attitudes and level of knowledge. However, the physical structure of the SFS compromises the adoption of good practices. It risks the safety of the food served to students at the evaluated public schools.

## Introduction

The Brazilian National School Feeding Program (Programa Nacional de alimentação escolar—PNAE) is historically one of the oldest programs in the country as a public policy for Food and Nutrition Security (FNS), created in the 1950s as a strategy to fight endemic hunger (1). The objective of the PNAE is to contribute to the health, growth, biopsychosocial development, learning, school performance, and healthy eating habits of low-income students with the use of food and nutrition education strategies and the provision of meals that cover their nutritional needs during the school year. Food hygiene and sanitation should be implemented in the PNAE by adopting good food hygiene practices and the continuous training of food handlers.

Considering these assumptions, good practices will be compromised and inevitable due to the precarious physical structure, the absence of standardized operating procedures, and the flow crossing in food production. In the same direction, in a vicious cycle, the health of schoolchildren will be compromised due to Foodborne Diseases. Health authorities have reported the occurrence of food outbreaks in public schools in several countries (2, 3). Traditionally, in Brazil, the public education system population has the lowest income, compromising access to food and health. Vulnerable populations, such as schoolchildren, need schools with a satisfactory sanitary classification to offer safe food and reduce foodborne diseases (FBD). FBD compromises children's health and consumes family resources for treatment.

Therefore, the hygienic-sanitary quality of meals is necessary. This will be ensured in the school program by adopting good food handling practices and by training food handlers (4). Strategies are used to identify food handlers' level of knowledge, attitudes, and hygiene practice (5–8). Assessing the food safety knowledge and practices of food handlers is essential for improving food safety to prevent FBD (9). Increasing food safety knowledge improves the control and regulation of FBD (9).

Training in food hygiene will allow knowledge acquisition that may change attitudes and work practices and contribute to adopting good hygiene practices (9). Also, training has been proved to improve the knowledge of food handlers, comparing knowledge before and after training (5). Besides the training of food handlers, there are limiting factors for adopting good hygiene practices in public schools, such as: layout that favors flow crossing, areas with high temperatures, high humidity, buildings (eg layout that favors cross-flow, areas with high temperatures, high humidity), equipment, and utensils (eg, poor hygiene conditions, insufficient size, and number); the absence of uniforms, and inadequate environmental hygiene (for example: use of uniforms that handlers come from public transport or use of inappropriate clothing such as shorts).

Some studies tried to show how different strategies may improve handlers' knowledge and attitudes toward better hygiene in meal production. A study was conducted with food handlers in Community Restaurants (CR) in the Federal District (10). The results showed that after the educational campaign, there was a significant change for better design and execution of good manufacturing practices (10). A cross-sectional intervention study in restaurants evaluated the knowledge of food handlers in two stages (after and before training) (11). The lowest level of knowledge was identified concerning food storage conditions. Only 15.6% of study participants knew frozen food does not eliminate the potential risk due to various microorganisms. After training, correct answers increased, showing a significant difference. The authors conclude that continuing education of food handlers can significantly affect the number of foodborne illnesses and, consequently, better public health protection. It is noteworthy that several studies analyze both the effect of training in different types of restaurants around the world (12–14) and the physical structure and good manufacturing practices according to Brazilian legislation (15) for food service and school food services (12–14, 16).

Other strategies have been adopted to verify the relationships between attitudes, knowledge, and measures of adequate practices. A study with food handlers built an instrument using the knowledge, attitude, and practice model (KAP). It demonstrated that knowledge is important in fighting foodborne disease outbreaks, and adequate food safety knowledge will help the positive mood of handlers for food safety and will ensure that they are well motivated to practice hygiene (14). Another study with the same food handlers identified that food safety knowledge positively impacts food handlers' attitudes to their adherence to hygienic-sanitary conditions for food safety (17). Further, other than the indirect effect of the handler's attitude on food safety knowledge and personal hygiene, food handlers' attitude partially mediates the impact of knowledge on kitchen hygiene and disease control measure. It is noteworthy the innovative character of the study design and that researchers can adopt such strategies.

All the highlighted studies show that somehow researchers continue to seek answers to the precariousness in the production of meals offered to low-income schoolchildren. In the case of Brazil, seeking accurate information about meal production conditions is even more important if such information serves as a basis for developing intervention strategies and action plans, indispensable precursors for change. However, there must be reliable information to start such a process. Brazil lacks an expanded information system about the country's schools. That is why it is so necessary that we obtain accurate information that supports us in fighting for changes with the government. We are a continental country; for example, our HDI varies between 0.454 and 0.891 (18). The northeast of Brazil, where Salvador is located, has the worst indicators in the country. Given the magnitude of the PNAE and its importance to the nutrition and well-being of children from low-income communities, this study aims to identify factors that interfere with the adoption of good hygiene practices in public school food services in Bahia, Brazil.

## Materials and methods

This study is part of the Research Project entitled “Occupational Risks in School Food Services,” approved by the Research Ethics Committee of the School of Nutrition of the Federal University of Bahia (protocol no. 2121882).

This cross-sectional, exploratory study was applied in public schools in a county in Bahia/Brazil. It included (i) evaluation of Good Hygienic Practices in School Food Service (GHPSF) (19, 20); (ii) Identification of physical areas in foodservice schools and environmental comfort measures; (iii) identification of sociodemographic and occupational characteristics and attitudes assessment and level of knowledge in food hygiene. Data collection took place from August 2018 to August 2019. After the initial construction and evaluation by the judges, the instruments were applied to eight food handlers in three public schools to assess the understanding and relevance of the items. Such results were not included in the study sample.

### Sampling of school food service

The city of Salvador has 11 school districts and a total of 434 schools. Such numbers were informed by the Department of Education of the city of Salvador-Bahia-Brasil. The minimum sample of 158 schools (confidence level of 95% and an error of 5%) was stratified considering the total number of districts (areas) and the number of schools in each of the areas using the Excel® 2005 program for Windows version 10. All schools were included in the random draw (each received a number before the draw). If the school principal did not agree to participate in the study, a replacement school was picked from another draw and invited to participate. Only three school principals did not agree to participate, and all the subsequent principals agreed to participate in the study. The average number of students in public schools participating in the study was 363.1 ± 200, with a minimum number of 76 and a maximum of 1,211. Regarding food handlers, the minimum number per evaluated public school was 1, and the maximum number was 5 (an average of 2 food handlers per SFS).

### Evaluation of good hygienic practices in school food services

The checklist Good Hygienic Practices in School Feeding (GHPSF) (19, 20) was used to evaluate the adoption of good hygiene practices. For the analysis of the GHPSF checklist in the school food services (SFS), two criteria were adopted:

(a) Assessment of SFS using the GHPSF checklist as proposed by the National Education Development Fund (Fundo Nacional de Desenvolvimento da Educação—FNDE) (19, 20). Sensitivity and specificity were estimated using the Receiver Operating Characteristics (ROC) curve, and the checklist was evaluated using the Likert scale. After applying the checklists in schools, the area under the curve value of 0.79 was found for the SS-196 checklist and 0.85 for the 542/2006 administrative checklist. These values indicate good adequacy of the GHPSF checklist to identify inadequacies. The evaluation is based on the grade assigned to each item, the grade of the thematic block according to the weight and constant (K), and the final grade obtained considering the sum of the grades of the thematic blocks.

The GHPSF checklist is composed of 112 items categorized into six thematic blocks: (1) Buildings and facilities in the food preparation area with 36 items and a statistical weight of 10; (2) Equipment for controlled temperature with 36 items and a statistical weight of 15; (3) Food handlers with eight items and a statistical weight of 25; (4) Food inbound with four items and statistical weight of 10; (5) Processes and productions with 35 items and a statistical weight of 30; (6) Environmental hygiene with 20 items and statistical weight of 10. The Brazilian documents used to determine the blocks are based on international documents from Codex Alimentarius and World Health Organization (21, 22). Some examples of the items evaluated in each block are: Block 1—unit location, floor, and wall types, ceiling conditions, door and windows' materials, and conditions; Block 2—the presence of freezers, fridges and thermometers, heat counters, control of temperatures for meats; Block 3—all handlers use uniform, periodic health exams, protected hair; Block 4—packing control of received food, control of expiring date, control of food characteristics upon receiving; Block 5—hand hygiene, food storage, food labeling, defrosting in adequate temperature; Block 6—garbage area is isolated; garbage is taken out daily; the hygiene products are registered.

For the analysis, we used the equation proposed by the FNDE:

(1) $$\begin{array}{l} Block\;Punctuation\\ =\;\frac{\text{Total of “yes” responses}}{\text{Total of items in the block }-\text{ Total of “not applicable” }}\\ \times \;Block\;weight \end{array}$$

Considering:

*Block punctuation* = *total score per thematic block*.

*Total of “yes” responses* = *sum of the number of responses marked as “yes” in each item of the checklist*.

*Total of item in the block* = *sum of items in the block*.

*Total of “not applicable”* = *sum of the number of responses marked as “not applicable” in each item of the checklist*.

*Block weight* = *the degree of risk of the situations (or conditions) belonging to this thematic block. The weight consists of a constant value, whose sum resulted in 100, and which acts as a multiplier in the blocks, giving higher scores to the items considered to be of greater risk*.

The overall SFS sanitary classification was based on the mean of the scores of the six blocks, and the result was presented in adequacy percentage. The block punctuation was used to calculate the score for each block per school. The GHPSF checklist (19, 20) classifies the SFS according to the obtained score in Very High Health Risk situation (0–25%); High Health Risk situation (26–50%); Regular Health Risk (51–75%); Low Health Risk (76–90%) and Very Low Health Risk (91–100%). Compliance is the proper fulfillment of each of the items. The total of items properly completed was converted into a percentage for later risk classification. Classification of the SFS followed the parameter described by Stedefeldt et al. (20).

### Identification of the SFS physical areas and environmental comfort measures

With the making of the sketch, which included all the areas for reception, storage and handling of food, a technical visit was carried out to evaluate the spatial conditions (measurement of spaces and conservation conditions), measures of environmental comfort and food safety in the SFS. The physical and technical barriers were observed (identification of the location of each task in the physical space). This evaluation considered what was proposed by the GHPSF Checklist (raw material storage, cafeteria/canteen, cleaning material storage area) (20) and Resolution 216/2004 (food inbound and pre-preparation of meat) (23). Also, for the spatial conditions and the environmental comfort conditions, the parameters of NR 15 (exposure to heat at a temperature of up to 26.7°C, in a journey of up to 8 h) (24) and NR 17 (the minimum humidity limit <40%) (25) were considered.

These measurements were collected because environmental temperature and humidity are extrinsic factors that corroborate bacterial multiplication. The measurements were performed in triplicate, with an interval of 1 min. The area of each SFS was calculated (Equation 2). Researchers used the globe thermometer of the wet bulb (TGBU, Minipa®), calibrated according to Resolution no. 029/95 of the National Institute of Metrology, Quality and Technology (INMETRO) (26). Data collection occurred in the morning, at reception and storage of foods, pre-preparation of meat, cafeteria/dining room/distribution of meals, and production of food SFS area that had physical separation. When physical separations did not exist, measurements were carried out in the available physical space. Measurements occurred in August 2018 for part of the schools and August 2019 for the rest of the sample.


(2) $$\begin{array}{l} Average\;of\;each\;school\;foodservice\;area=width\;\times lenght \end{array}$$

### Evaluating of the characteristics, attitudes and knowledge of food handlers

The sample calculation at this stage considered the number of food handlers (*n* = 318) working in the 159 schools included in the study, using a confidence interval of 95%, and a sampling error of 5%. Therefore, the minimum sample calculated was 139 food handlers. All of the 318 food handlers were invited to participate. A total of 142 food handlers were evaluated at the end of the process since many refused to participate.

A previously validated semi-structured questionnaire identified sociodemographic and work characteristics, attitudes, and knowledge levels (19). The questionnaire consisted of 49 items, distributed in thematic blocks: (1) socioeconomic characteristics with four items (age, sex, education, and salary); (2) information on the work routine with 12 items (type of employment—civil servant or with a contract, working time in school meals, weekly working hours, function registered in the work and employment card—CTPS, intermittent contract, participation in training on food hygiene); (3) knowledge, with 14 adopting dichotomous responses: correct or incorrect; (4) attitudes, with 19 items adopting a five-point Likert scale ranging from (1) Strongly disagree, (2) Partially disagree, (3) I neither agree nor disagree, (4) I partially agree, (5) I totally agree. The data obtained per block on the level of knowledge and attitudes were transformed into percentages (score) using the following Equations 3 and 4, respectively:


(3) $$\begin{array}{l} \text{Level of knowledge}=\frac{\text{number of items assigned as “correct”}}{\text{total number of items of thematic block}}\\ \;\times 100 \end{array}$$

(4) $$\begin{array}{l} \text{Level of attitudes}=\frac{\left(\frac{\text{sum of likert responses}}{\text{total number of item of thematic block }}\right)-1}{5-1}\\ \;\times 100 \end{array}$$

The analysis by the thematic block made it possible to identify attitudes and the level of knowledge in adopting good hygiene practices in school feeding. The classification of the thematic blocks was based on the obtained percentage: Unsatisfactory (0–49.9%); Satisfactory with Restriction (50–69.9%), and Satisfactory (≥70%) (27, 28). In addition, these scores were compared considering sociodemographic and work characteristics.

### Statistical analysis

The data were analyzed using the Statistical Package for Science—SPSS® in version 26.0. The categorical variables were described as frequencies and percentages, and the quantitative variables as mean and standard deviation. The comparisons of levels of knowledge and attitude by socioeconomic and demographic characteristics were performed by independent Student *t-*test in cases where two groups are compared. In those cases where there are three or more groups of comparisons, a one-way Analysis of variance (ANOVA) followed by Tukey's *post-hoc* test was performed. All tests were performed considering bilateral hypotheses and a 5% significance level.

## Results

### Evaluation of good hygienic practices in school food services

Table 1 presents the characteristics of the SFS by the block of the Good Hygiene Practices in School Feeding checklist in a county in the state of Bahia. The classification of the SFS sanitary status, using the recommendations of the FNDE from the GHPSF checklist, was of moderate risk in 74.8% (*n* = 119) of the SFS (51–75% of compliance) and 25.2% (*n* = 40) at high risk (26–50% compliance). Conformity for the 159 SFS was 50.23%, obtaining a high-risk classification (26–50% compliance).

**Table 1:** The sanitary conformity of different areas of the food service facilities of the schools.

**Block**	**Mean (SD)**	**Max. score**	**% Conformity**	**Sanitary risk classification**
1. Buildings and facilities in the food preparation area	42.23 (9.49)	91	50.62%	Moderate
2. Equipment for controlled temperature	10.92 (7.92)	68	18.19%	Very high
3. Food handlers	19.02 (4.41)	26	79.18%.	Low
4. Food Inbound	21.37 (2.96)	22	97.14%	Very low
5. Processes and food productions	72.94 (21.65)	201	49.69%	High
6. Hygiene Area	41.03 (10.73)	84	79.40%	Low
Good Hygiene Practices in school feeding	41.65 (9.20)	82	50.23%	High

The blocks that obtained means classified as high sanitary risk were 1 and 5. The nonconformities were (i) block 1: floors, walls, doors, and windows in precarious conditions, inadequate conservation and covering materials; absence of millimeter screens and exclusive washbasins for the hands' hygiene and (ii) block 5: inadequate or non-existent hands' hygiene of the food handlers; inadequate food storage after the pre-preparation process; incorrect disinfection (sanitization) procedures for vegetables and fruits; absence of a manual of good hygiene practices and Standard Operating Procedures (SOP). In block 2, classified as very high sanitary risk, the following nonconformities were identified: insufficient equipment; inadequate state of conservation and hygiene of equipment; absence of temperature control in the cold and hot chain.

Blocks 3 and 6 were classified as low sanitary risk and presented nonconformities. In block 3, the absence of a complete uniform for food handlers was identified; use of adornments and non-performance of periodic examinations and in block 6, the lack of an exclusive area for the storage of waste; the presence of vectors and urban pests; error in the chemical disinfection processes of the utensils and inadequate hygiene conditions of the “cleaning tissues”. Block 4 was classified as a very low sanitary risk. However, there was a lack of evaluation of the integrity of the food packaging and a lack of return of inadequate raw material at the time of inbound.

### Compliance with food service facilities

Table 2 presents missing areas in the SFS facilities according to the GHPSF checklist. The absence of storage areas for dry food, meat preparation, and trash storage in more than 98% of schools was observed in the SFS.

**Table 2:** The compliance of School Food Service facilities of schools in Bahia/Brazil.

**Missing areas**	**Number of school food services with missing area**	**% of schools with missing areas**
Goods inbound area	158	99.4%
Dry food storage	37	23.3%
Meat pre-preparation area	159	100%
Canteen	88	55.3%
Cleaning material storage	58	36.5%
Trash storage	156	98.1%

### An evaluation of the food safety attitudes and level of knowledge of food handlers

A total of 142 food handlers agreed to participate in this study phase. Most were female (*n* = 140; 98.6%), mean age was 46.8, with complete high school (*n* = 68; 47.9%), receiving 1 minimum wage (*n* = 120; 84.5%); working in school foodservice up to 5 years (*n* = 72; 50.7%) and most of them also participated in food hygiene training (*n* = 123; 86.6%) (Supplementary Table S1).

For food safety attitudes, 1.4% (*n* = 2) of food handlers were classified as Unsatisfactory (0 to 49% of correct answers), 8.5% (*n* = 12) as Satisfactory with Restriction (50–69% of correct answers) and 90.1% (*n* = 128) as Satisfactory. Most of them reported: wearing a full uniform during food preparation and distribution (69%; *n* = 98), separating raw and cooked foods during storage (88%; *n* = 125), and hand hygiene before handling cooked food (96.5%; *n* = 137). Comparing the attitude and socioeconomic and demographic variables, there was no significant difference for age (0.296), educational level (*p* = 0.597), mean wage (*p* = 0.652), or time working in school foodservice (*p* = 0.120). However, it was significant for the weekly workday (*p* = 0.044) and participation in food hygiene training (*p* = 0.008) (Table 3).

**Table 3:** Mean and standard deviation of attitude and knowledge scores on Good Hygiene Practices of food handlers in School Food Services with different socioeconomic and demographic characteristics.

	**Knowledge**		**Attitude**	
	**Mean (SD)**	***p***	**Mean (SD)**	***p***
**Age**				
Up to 39 y/o (*n =* 34)	69.49 (15.62)		78.48 (15.13)	
40 to 49 y/o (*n =* 47)	70.66 (16.57)	0.172^*^	81.41 (11.02)	0.296^*^
50 y/o or more (*n =* 61)	74.92 (13.51)		82.40 (10.07)	
**Schooling**				
Elementary School(*n =* 70)	70.24 (16.68)	0.128^**^	80.60 (11.28)	0.597^**^
High School (*n =* 72)	74.12 (13.37)		81.65 (12.30)	
**Mean wage**				
Up to 1 MW (*n =* 125)	71.48 (15.53)	0.121^**^	80.97 (11.99)	0.651^**^
More than 1 MW (*n =* 17)	77.56 (11.08)		82.35 (10.35)	
**Time working in school food service**				
Less than 1 year (*n =* 17)	67.90 (14.50)		80.19 (8.01)	
1-5 years (*n =* 55)	75.19 (13.91)	0.194^*^	83.16 (12.91)	0.120^*^
5-10 years (*n =* 24)	68.81 (15.83)		76.32 (13.97)	
11 years or more (*n =* 46)	72.02 (16.18)		81.58 (9.72)	
**Weekly workday**				
20-30 hours (*n =* 100)	71.07 (14.29)	0.167^**^	79.89 (12.02)	0.044^**^
40 hours (*n =* 42)	74.93 (16.95)		84.09 (10.75)	
**Participation in food hygiene training**				
No (*n =* 19)	67.43 (16.66)	0.141^**^	74.51 (17.39)	0.008^**^
Yes (*n =* 123)	72.95 (14.86)		82.16 (10.39)	

* One-way ANOVA with Tukey's *post-hoc* test.

** Independent Student t-test.

There was no significant difference between the level of knowledge (Table 3) and socioeconomic and demographic characteristics (*p* > 0.05). A significant different was observed in attitude when food handlers were separated by weekly workday and participation in food hygiene training.

Table 4 presents the number and percentage of knowledge level for each item within the instrument about knowledge of Good Practices. For knowledge, the lowest score was “Contaminated food will always have some change in color, smell, or taste, and the highest was “To avoid food contamination, I wash and disinfect my hands before preparing food”.

**Table 4:** The knowledge of food handlers in the School Food Services on Good Hygiene Practices'.

**Items**	**Score**
**Knowledge**	
Q1. Hot foods prepared in the morning can be left in the kitchen at room temperature to be served on the next meal.	68.3
Q2. I know or have heard about the diseases: salmonellosis, shigellosis, botulism, staphylococcosis, cholera, and hepatitis A.	52.8
Q3. Among the symptoms of intestinal infection are fever, stomach pain, diarrhea, vomiting, and nausea.	88.0
Q4. To avoid food contamination, I wash and disinfect my hands before preparing food.	96.5
Q5. A thermometer is used to know the temperature of ready-to-eat foods in the school food service.	73.2
Q6. School food service should only be cleaned at the end of the day.	81.0
Q7. Meat can be defrosted in a bowl of water outside the refrigerator or in the sun, or at room temperature.	65.5
Q8. Contaminated food will always have some change in color, smell, or taste.	6.3
Q9. Hiring a company to kill rats and insects in the school food service is necessary.	94.4
Q10. Vegetables, fruits, and meats can be cut on the same cutting board or plastic plate.	72.5
Q11. It is necessary to put the expiration date on food packages that have been opened but not fully used.	83.8
Q12. School food handlers must perform blood, stool, and urine tests every year.	91.5
Q13. The drying of utensils must be carried out with dishcloths.	52.1
Q14. Filtered water is used to prepare juices and wash fruits.	86.6
Total	72.2

The most reported practices were the use of different utensils for the pre-preparation of vegetables, fruits, and meats by 72.5% (*n* = 103) of food handlers, control of food temperature with a thermometer (73.2%; *n* = 104), and performance of periodic examinations by the food handlers (91.5%; *n* = 130). The items with the highest number of errors performed by the food handlers were food remaining at room temperature for a period longer than 2 h (31.7%; *n* = 35); identification of the names of foodborne diseases (47%; *n* = 67), and sensory changes in contaminated foods (color, odor, and taste) (81%; *n* = 115) (Table 4). The percentage of correct knowledge answers of food handlers was 81.13%, characterizing the knowledge of food handlers as satisfactory (≥70%), with 96.5% (*n* = 137) stating that “To avoid food contamination, I wash and disinfect my hands before preparing food”.

For attitude, the lowest score was related to washing the fruit with water and vinegar (2.60), and the highest score was related to procedures for verifying the integrity of food packaging (4.98) (Table 5).

**Table 5:** Mean (M) and standard deviation (SD) attitude according to food handlers in the School Food Services (based on 5-point Likert scale responses).

**Attitudes**	***M* SD**
Q1. I must learn the procedures to avoid food contamination.	4.85 (0.67)
Q2. I must clean benches or worktables at the end of each shift to prevent food contamination.	4.62 (1.12)
Q3. I can store ready-to-eat foods with raw foods.	4.60 (1.13)
Q4. I can store food in the pantry inside cardboard boxes and plastic bags to avoid contamination from rats and insects.	4.61 (1.48)
Q5. I must clean the trash every day.	4.85 (0.71)
Q6. I should wash the fruit with water and vinegar to kill the germs.	2.60 (1.87)
Q7. I believe that cooked food cannot be contaminated.	3.57 (1.79)
Q8. I can store food with cleaning products that do not have an odor.	4.72 (0.99)
Q9. I wash my hands before touching raw food.	4.85 (0.65)
Q10. I always wash my hands before touching cooked food.	4.94 (0.37)
Q11. I always wash my hands before going to the bathroom.	3.83 (1.71)
Q12. I always wash my hands after using the bathroom.	4.91 (0.52)
Q13. I wear the full uniform when I prepare or distribute food.	4.12 (1.53)
Q14. I read food labels before preparing them.	4.56 (1.02)
Q15.I read the shelf life of foods before preparing them.	4.92 (0.42)
Q16. I check to see if food packages are torn/open before I prepare them.	4.98 (0.14)
Q 17. I use sodium hypochlorite (bleach) to wash the fruits.	4.6 (1.08)
Q18. I can refreeze food that has been thawed.	4.52 (1.08)
Q19. I talk to my colleagues during food preparation.	3.89 (1.54)
Total	4.42 (0.32)

It is noteworthy that food handlers have an extremely positive attitude score considering that the range of the scale used in this study is from 1 to 5 and that the average reached was 4.42 (sd = 0.32).

## Discussion

The 2021 Brazilian School Census (29) recorded 7.8 million enrolled students, of which the municipality served 49.6%. Therefore, investments in the physical structure, equipment, and food handlers training are essential to guarantee sanitary food quality for 3.86 million students during the 200 school days determined by Brazilian legislation (30).

Vulnerable populations, such as students enrolled in public schools (31), need schools with a situation of Low Health Risk or Very Low Health Risk to offer safe food and consequently reduce the occurrence of FBD. However, in this study, SFS were classified as High Health Risk and Regular Health Risk, similarly to other studies (13, 32–35), which compromises food safety and the health of schoolchildren. In addition to compromising children's health, foodborne diseases consume family resources for their treatment. It is necessary to invest in the physical structure and equipment of the SFS as a way to minimize cross-contamination of food and provide decent conditions for the consumption of meals (36, 37).

The assessment of sanitary conditions in SFS using checklists is a low-cost, and easy-to-apply by professionals trained for this purpose. Different checklists were used in studies to diagnose sanitary conditions and reassess the adoption of corrective measures in SFS (32–34). Four studies evaluated the sanitary conditions of public schools in different states and counties in Brazil. Cardoso et al. (32) and Gomes et al. (33) used a checklist prepared with RDC 216/2004 (23) to evaluate the 235 public schools in Bahia and 18 schools in Goiás, respectively. Ribeiro et al. (38) and Soares et al. (34) used the LVGHPSF (39) in six schools in Vale do Ribeira, São Paulo, and nine schools in a city in Rio de Janeiro, respectively, the same instrument applied in this study. The SFS participating in this study were classified as High Health Risk and Regular Health Risk (39). The results were similar to those found in the studies by Cardoso et al. (32), Gomes et al. (33), and Ribeiro et al. (38) and different from the study by Soares et al. (29). They found one school with a High Sanitary Risk, six Regular Sanitary Risk, and two with Low Sanitary Risk in Rio de Janeiro. The SFS that presented the classification of Low Sanitary Risk had better scores for Buildings and Installations and Equipment for controlled temperature.

The evaluation of the public SFS and the working conditions of food handlers in Brazil are the object of study by several researchers. In general, public SFS do not comply with the FNDE (40). This situation has been maintained over the years in a demonstration that greater investment by the government is necessary for this area.

The sanitary nonconformities presented in the SFS favor the preparation of meals that diverge from the objective of the PNAE: growth and biopsychosocial development of schoolchildren and the formation of healthy eating habits (40), as it jeopardizes the production of safe food and, consequently, the occurrence of food outbreaks. In this context, the county must invest in restructuring Food Services to produce safe food in all schools, using the principle of equity proposed by the Brazilian Organic Health Law (41).

When analyzing the blocks, differences in the Health Risk Situation are identified. In block 1 (Buildings and Installations in the Production Area), the results of this study differ from other studies (32, 34). In the first study (32), the authors identified that 56.2% of the SFS presented compliance, and in the second (34), the SFS presented results between 58.1 and 73.7%, with this block classified as a regular risk. Therefore, the building conditions found in the SFS by these authors are better than those in our study. Nunes et al. (35), evaluating GMP in 13 elementary schools in Taquari, Rio Grande do Sul (Brazil), identified that the factors that interfered with the block 1 result were lack of protection of light, walls, and ceilings that were difficult to clean, doors without automatic closing, windows without pest protection screens and disordered production flow, with a score of 69.54 ± 4.40%. Lemos et al. (36), investigating GMP in elementary schools and daycare centers in the county of Madalena, state of Ceará/Brazil, and found a percentage of 61.8% for block 1. The score in this study was lower, but the factors that interfered with the score are similar to those cited by other authors and put food safety at risk and, consequently, the health of schoolchildren.

Block 5 (Processes and productions) addresses issues related to health documents: good hygiene practices manual and standard operating procedures (SOP). These documents proposed in RDC 216/2004 (23) are vital for the training stages of food handlers and for monitoring the SFS. These documents do not indicate that such procedures are carried out or monitored. However, the absence indicates the impossibility of execution and monitoring by food handlers.

Studies (13, 32) identified the absence of these documents in the SFS, data similar to our study. One study (42) identified different results that evaluated the sanitary conditions in 12 SFS in Rio Grande do Sul/Brazil. The authors reported that the SFS had good hygiene practices manual and SOP, but the documents were not available to the food handlers. Another study (43) identified 42.85% of SFS with good hygiene practices manual. Documents must be available for handlers to verify that activities are carried out with proper procedures. Other items evaluated in block 5 were food thawing and hand hygiene. In this study, most SFS adopted the thawing of food at room temperature, and during the visual observation of the researchers, hand hygiene by food handlers was not identified. Thawing at room temperature or meats immersed in water without checking the temperature were data identified by Cardoso et al. (32) in 68.9% of the schools. These procedures favor bacterial growth and increase the risk of cross-contamination and foodborne disease (44).

The lack of hand hygiene in SFS is possibly related to the absence of exclusive washbasins, antiseptic soap, and non-recycled paper towels (23). This data was also observed in another study (33). The authors consider that the physical-functional structure associated with hand hygiene is a fundamental factor for not adopting protective hygiene habits. A study (45) suggests the need for greater involvement of managers for the adequacy of SFS as provided in the current health legislation (23).

Block 2 (Equipment for controlled temperature) received the lowest score among the evaluated blocks. It was identified that the absolute majority of schools did not perform temperature control in the cold chain or the hot chain. This event is due to the lack of simple equipment such as a thermometer (100%). In addition, it must be considered that these SFS do not have a sufficient number of refrigerators. When present, they are in an inadequate state of conservation (<60%), and there is not the presence of pass-through or thermal distribution counters that guarantee compliance with the time/temperature binomial (23) Some conditioning factors for foodborne diseases are time and temperature control failures, poor environmental hygiene, and inadequate food handling. These factors favor bacterial multiplication and may cause food outbreaks in public schools. In Brazil, from 2000 to 2017, 8.6% of the reported outbreaks occurred in schools (46).

Despite the sufficient classification of attitudes and knowledge of food handlers, the lack of a thermal counter for distributing food to schoolchildren led food handlers to portion the preparations in utensils (cups and/or plates), distributing them directly to schoolchildren to be consumed in cafeterias and/or patios and/or corridors or direct to classrooms for consumption. However, the time and temperature binomial criteria were not adopted in the distribution process. According to RDC 216/2004 (23), food may remain at room temperature for up to 2 h, as the multiplication of bacteria during this period would not favor the occurrence of FBD (44). A study showed a similar result, identifying the absence of cafeterias in 74.5% of the SFS, with food consumed in the courtyards or the classroom (32). The patios are often open areas without washbasins for hand hygiene, with objects in disuse, and the presence of animals. These data are similar to the studies by Cardoso et al. (32) and Lemos et al. (13). The first (32) identified that 99.1% of SFS did not have equipment for hot storage of ready-to-eat food, and maintenance was at room temperature. The second (13) verified that there was no measurement of temperatures during the distribution of school meals in the visited schools, and the distribution took place immediately after preparation.

Blocks 3 (Handlers) and 6 (Environmental Hygiene) received the low-risk classification, but nonconformities were also found. In block 3, the use of incomplete uniforms and adornments can be seen, diverging from the recommendations of the current legislation RDC 216/2004 (23) guides employers to provide enough uniforms for daily change (23). Another study (43) identified a different percentage of compliance, 71.78%, but the evaluated items were similar to our study. In our study, the item with the highest percentage of compliance was using caps, which kept the hair wholly protected. This result is similar to that identified by a study (32) in which food handlers did not use their uniforms properly, but 66.0% of the handlers had their hair wholly protected. Another study (33) found that personal hygiene improved after handlers participated in food hygiene training (*p* < 0.05). In this sense, several authors have recommended the adoption of active methodologies as a way to improve the adoption of good practices (6, 10).

Periodic examinations by food handlers are a sanitary requirement to prevent FBD during food handling (23). The SFS food handlers did not perform periodic examinations. A study (13) found that 60% of SFS did not have a health control program for handlers, and another study (47) showed that 55.9% of handlers from 35 public schools in Tocantins/Brazil did not perform periodic exams.

In Block 6 (Environmental hygiene), household cleaning products were identified for cleaning areas with a volume of food production compatible with medium and large SFS, considering the number of meals (48). These are not suitable for the sanitizing process of semi-industrial equipment. Another aggravating factor is the absence of standardized operational hygiene procedures (SSOP) to instruct food handlers on the proper chemical disinfection of utensils and equipment, sponges for washing utensils, and non-disposable cleaning tissues. The absence of SSOP may favor the inadequate dilution of detergents and bleach, with an active chlorine concentration of 2–2.5%, commonly used in SFS, allowing the formation of bacterial biofilms (49) and contamination of the surfaces of utensils, and equipment, and consequently of food, putting food safety at risk (50).

A study (51) found differences in hygiene conditions between the first and last visits in cleaning equipment, furniture, and utensils at SFS. The authors attributed this result to the doubts that the researchers clarified to the food handlers during the technical visits: correct cleaning of the place of manipulation, filling in SSOP worksheets, and sanitization of facilities, equipment, and utensils. This result corroborates the need to implement correct cleaning procedures, constant supervision in the SFS, and corrections of the handlers' procedures.

Another nonconformity identified in block 6 was the presence of vectors and urban pests in SFS. It may be related to the lack of protective screens on doors and windows and other control measures to prevent access, shelter, and pests' proliferation. This data differs from the study by Lemos et al. (36), who classified SFS as “Poor” [20 to 49% adequacy of RDC 216/2004 requirements (16)] and “Very Bad” [0 to 19% adequacy of RDC 216/2004 requirements (16)], respectively, regarding integrated pest control. It is important to highlight that most schools presented certificates of chemical control and disinsection at data collection.

Block 4 presented the best sanitary classification among the blocks. However, it is limited to four items related to the raw material: sensory characteristics, packaging integrity, return of disapproved products, and expiration date on food labels. Block 4 does not evaluate the hygiene conditions of the vehicle and delivery people, temperature control, and hygiene of the reception area, as recommended by RDC 216/2006 (23). Most pathogenic microorganisms do not change the sensory characteristics of food (50). Thus, adopting these control measures avoids receiving contaminated raw material and the risk to the health of schoolchildren. The analysis by block allowed us to identify which items influenced the sanitary risk situation in the SFS and which need the adoption of corrective actions to protect the health of schoolchildren.

### Compliance with food service facilities

The absence of physical areas assessment contributes to analyzing the flow crossing of food production, people, and waste (48). Thus, this study evaluated the absence of areas for reception, storage, pre-preparation of meat, cafeteria/dining room, cleaning products, and waste storage at SFS. It is important to emphasize that the inadequate physical structure due to the precarious conditions of conservation, the absence of areas, and errors in the routines of the food handlers interfere with the linear flow. It results in poor hygiene of the environment. It favors the attraction/shelter/proliferation of vectors and urban pests and consequently exposes food to the risk of food contamination (50) and work accidents involving handlers.

In this study, activities related to food production took place in a single space, with crossing flows and the absence of physical barriers that favored cross-contamination of food. These data were similar to a study (32) that identified that 50.2% of the SFS did not have a linear and unidirectional flow due to the production of meals taking place in a single space, which compromised food safety and food and nutrition security, as recommended by the PNAE (46). Most SFS had an exclusive area for the storage of dry food. However, the physical spaces were insufficient for the adequate disposal of raw materials to favor air circulation, adequate stacking, and the proliferation of pests (23). These data are similar to a study (32) that identified in 81.7% of the SFS that non-perishable products were stored on shelves. However, in 84.7%, the food “was not far from the wall, floor and/or ceiling,” facilitating access by insects and changes in humidity. Which consequently can reduce the shelflife of the raw material.

The meat pre-preparation area was absent in all SFS. This data is similar to Cardoso et al. (27), who observed that 96.6% of SFS did not have separate areas for handling raw and cooked foods, contributing to cross-contamination. The World Health Organization suggests that raw and cooked foods are placed in separate areas to avoid food contamination (44). More than 50% of the SFS did not have a cafeteria/dining room for students to use during food consumption. Thus, school meals were consumed in classrooms, open areas, and other spaces where people, food, and waste circulated. This result is similar to another study (32) that identified the absence of canteens in 74.5% of SFS.

Due to the lack of an exclusive area for the storage of cleaning products, in many schools, these were stored in spaces intended to store school materials and/or in the food production area and/or in the dry pantry at the SFS. Storage of cleaning materials close to food favors chemical contamination. Chemical hazard is one of the causes of food poisoning (44). Waste storage, in most schools, took place in a physical space intended for the movement of people and food, as there was no specific area. The absence of an adequate area for storing waste favors the attraction and shelter of urban pests. It is important to emphasize that the containers used for storage were covered, and most had the lid activated by a pedal. Cardoso et al. (32) found a different percentage from this study because, according to the authors, inadequate waste storage occurred in only 42.6% of schools.

Regarding the evaluation of the adoption of good practices in the SFS, Soares et al. (34) evaluated that the presence of items such as a good practices manual, SOP, and control of the binomial time and temperature is far from the reality of SFS, as they have a physical structure of a “domestic kitchen”. The number of food handlers is often inadequate. In addition, the daily absence of a dietitian/nutritionist interferes with a more effective hygienic-sanitary control of the meal production process. This study emphasizes that institutional canteens and SFS must comply with the recommendations of RDC 216/2004 (23), not only for regulatory reasons but also because of the profile of the public served—infants, children, and the elderly, in conditions of social and economic vulnerability. Consequently, states and counties must invest in the physical-functional planning of SFS for the food and nutrition security of the public. Thus, school feeding is a public policy that favors access to food, and these must have adequate sanitary characteristics.

#### An evaluation of the food safety attitudes and level of knowledge of food handlers

Training of food handlers on topics related to food hygiene is necessary for the implementation of good food manufacturing practices. The objective is to allow them to practice and produce safe food. For practices appropriate to health legislation, handlers must have attitudes and knowledge that direct them to adoption. In this study, most handlers reported participating in food hygiene training. Other studies found different data (9, 27, 41), which identified that 80.9% of schools did not carry out the semiannual training of handlers, 26.7% of handlers claimed to have never participated in the training, and 48% said they had not participated in the training, respectively.

Food handlers must be supervised and periodically trained in personal hygiene, hygienic food handling, and foodborne illness. Training is a requirement of current health legislation in Brazil (23). Such legislation requires proof from documentation (16). However, it is necessary to plan actions that allow the active participation of food handlers, developing critical thinking to adopt good practices given the responsibility they have assumed in food production (6, 10). Pagoto et al. (7) found correct answers for hand hygiene after using the toilets and handling garbage (98.7%), checking the validity and integrity of products (98.7%), and the importance of learning about safe food handling (97.3%). The study by Hossen et al. (52) evaluating the level of knowledge of 200 street food handlers in Bangladesh identified that only 33% had satisfactory attitudes about food safety. In this study, the answers given by food handlers emphasized washing hands by food handlers before starting to prepare meals. The percentage of correct knowledge of food handlers was 72.2%, characterizing it as satisfactory (≥70%). Satisfactory knowledge was similar to those found in other studies already mentioned (7). Such knowledge is apparently reflected in the attitude of the handlers of this and other studies. This is certainly an encouragement to produce safe food from the point of view of contamination.

Attitude is the state of a person that predisposes them to a favorable or unfavorable response to an object, person, or idea (53). Thus, responses related to attitudes were judged by food handlers according to cognitive and affective aspects related to food hygiene. Knowledge about the topic interferes with people's perception. However, there are differences between the desirable attitudes and the hygienic-sanitary reality identified in the SFS. Half of the sample of food handlers did not have a complete uniform, which would make them start work with the clothes they used during the journey from home to work. There was not enough equipment to store food in the cold chain, a lack of physical separation between areas to avoid cross-contamination of food, and the inexistence of an exclusive washbasin for hand hygiene in all SFS.

The study by Kwol et al. (17) analyzed the variables of knowledge, attitude, and practices in a mediational model and found a positive impact of knowledge about attitude, as expected. Attitudes have, in turn, a positive impact on adherence to hygiene conditions. These make one think about what strategies to adopt to improve the sanitary safety conditions of school meals. On the other hand, our study identified that schools in Salvador-Bahia have problems that precede our investigation of adequate practices. The conditions found by the GHPSF checklist give us the exact dimension of what our main problem is. How to improve adherence to appropriate practices if buildings and facilities in the food preparation area, equipment for controlled temperature and processes, and food productions have percentages far below the desired values. Therefore, before thinking about education strategies, not that these are not important, we must improve the structural conditions.

One study (32) found that 77% of handlers said they sanitized their hands when arriving at work. However, the same study observed that in 51.7% of SFS this practice was not performed before handling food and touching any other material. In the study by Moghnia et al. (54), 99% of handlers reported the presence of an exclusive sink for hand hygiene in the foodservice, 95.3% responded that they washed their hands before preparing food, and 91.1% sanitized their hands after handling the waste. However, the authors did not investigate whether practices were following knowledge. In this sense, knowledge must be associated with objective conditions of adequate practices based on physical structures that do not favor the crossing of the production flow and the existence of adequate facilities, training, and supervision.

It is important to highlight that there was only a significant relationship between Satisfactory attitudes and participation in training and weekly workday as a food handler. Training, in fact, seems to be the most appropriate conduct to change the behavior of food handlers, as attitude is the first step toward an effective and positive change in behavior. In addition, the other variable that interfered with the attitude was the weekly workday. In this sense, a greater number of worked hours interfered with the positive attitude of the handlers. A positive attitude of food handlers is significantly associated with training and the number of hours worked per week. In a review of other studies, Waddell and Burton (17) stated that work is generally good for physical health and mental well-being. Unemployment is associated with the opposite, and work can reverse the adverse health effects of unemployment.

The assertions related to knowledge with the highest scores were using different utensils for the pre-preparation of vegetables, fruits, and meats, controlling food temperature with a thermometer, and carrying out periodic examinations by the handlers. This information differs from the observations carried out in loco at the SFS during the study. It is suggested that the training adopt active methodologies based on the transmission of knowledge. The use of active methods in the training can favor satisfactory attitudes and knowledge and corroborate good sanitary practices (10). However, it is essential to offer working conditions for these changes to occur within the scope of the SFS of the PNAE.

The SFS food handlers are almost entirely women, which has several consequences. The participation of women in tasks considered feminine is already well known to researchers (55, 56). Such studies report poorly paid jobs with long working hours requiring little education and professional qualifications (55, 56). According to (60), women worldwide represent the largest portion of the poor (70%) and illiterate (65%) population. At work, due to the predominantly informal, precarious, and part-time occupation, they are assigned roles labeled as female but which have a domestic appearance. To a lesser extent, they perform command functions in a naturalized hierarchical dimension at work and in politics. According to Antunes and Druck (57), Krein and Castro (58), and Nogueira and Carvalho (59), when analyzing outsourcing linked to the issue of gender, women are subjected to more insecure working conditions, with higher turnover, low qualification requirements, repetitive work, non-compliance with rights and strong insertion in the service provider segment. These are factors that characterize the precariousness of this type of hiring.

This study has some limitations since it is a cross-section study, and the correlational results do not reveal causal relationships between the variables. The study was only conducted in Bahia, and it does not represent the country. Also, the applied checklist does not include sustainability parameters in its evaluation. Although data collection on environmental comfort was carried out in the morning, the thermal amplitude of the city of Salvador-Bahia is between 22 and 31°C, which does not compromise the findings.

## Conclusion

This study sought to identify the factors that interfere with good hygiene practices in Salvador-Bahia public schools. We adopted previously validated instruments (content and semantics validation). Such instruments pointed out that food safety conditions are significantly compromised both by the highly inadequate conditions of the SFS concerning the physical, functional, and food handling conditions. In addition, simple measures such as avoiding cross-contamination between food and waste from the change in the garbage collection time or even the purchase of pedal-operated garbage bins with lids would avoid the possibility of contamination.

In summary, the factors that would interfere with the adoption of good practices in the SFS participating in the study were: the absence of physical areas to avoid crossing the production flows of food, people, and waste; insufficient number and/or inadequate conditions of equipment for food storage in the cold chain; the absence of time and temperature control in the stages of receiving and distributing food; insufficient number of food handlers for the distribution of work activities and, consequently, reduction of work overload. Food handlers showed satisfactory attitudes and level of knowledge. However, the physical structure of the SFS compromises the adoption of good practices. It risks the safety of the food served to students from the evaluated public schools. It is also essential to highlight the need for training and monitoring of handlers to adopt good personal hygiene and food practices.

The results obtained for the schools in this study were used to develop the training strategies for handlers and served as a subsidy for elaborating an action plan to correct the most critical points identified. Among the critical points, there are: the recovery of the physical structure of the schools, with adequate dimensioning of the areas, intending to allow the unidirectional flow, avoiding the crossing of food, people, and waste, purchase of equipment for storage in the cold and hot chain, construction of cafeterias for children and adolescents, among others. In addition, it is necessary to adjust the number of handlers to meet the production of meals according to the number of students, the menu type, and the time they stay in the SFS.

## Data availability statement

The original contributions presented in the study are included in the article/Supplementary material, further inquiries can be directed to the corresponding author/s.

## Ethics statement

This study is part of the Research Project entitled Occupational Risks in School Food Services, approved by the Research Ethics Committee of the School of Nutrition of the Federal University of Bahia (protocol no. 2121882). The patients/participants provided their written informed consent to participate in this study.

## Author contributions

Conceptualization, methodology, formal analysis, and investigation: JF, MA, and RA. Validation: JF, MA, RA, and EN. Resources: RZ. Data curation: JF. Writing—original draft preparation: JF, RA, RZ, and RB. Writing—review and editing: JF, RA, RZ, RB, and AR. Visualization: JF, MA, RA, RZ, RB, and AR. Supervision: RA, AR, AA-M, and MN. Project administration: JF, AR, and HH. All authors have read and agreed to the published version of the manuscript.

## Conflict of interest

The authors declare that the research was conducted in the absence of any commercial or financial relationships that could be construed as a potential conflict of interest.

## Publisher's note

All claims expressed in this article are solely those of the authors and do not necessarily represent those of their affiliated organizations, or those of the publisher, the editors and the reviewers. Any product that may be evaluated in this article, or claim that may be made by its manufacturer, is not guaranteed or endorsed by the publisher.
