# Supplementary material for: Factors interfering with the adoption of good hygiene practices in public school food services in Bahia, Brazil
Source: Front Public Health. 2022 Sep 15;10:975140. doi: 10.3389/fpubh.2022.975140 (PMC9521275; doi:10.3389/fpubh.2022.975140)
Supplement: Supplementary file 1 [file Table_1.DOCX]

Supplementary Material

**Table S1**. Sociodemographic and occupational characteristics of food handlers, in percentages and number, Bahia/Brazil.

| **Characteristics** |  | **%** | **n** |
| --- | --- | --- | --- |
| **Gender** | Female | 98.6 | 140 |
|  | Male | 1.4 | 2 |
| **Age** | 18- 29 y/o | 4.2 | 6 |
|  | 30-39 y/o | 19.7 | 28 |
|  | 40-49 y/o | 33.1 | 47 |
|  | 50-59 y/o | 33.8 | 48 |
|  | > 60 y/o | 9.2 | 13 |
| **Educational level** | Elementary school | 13.4 | 19 |
|  | Complete Middle school | 35.9 | 51 |
|  | Complete High school | 47.9 | 68 |
|  | Undergraduate | 2.8 | 4 |
| **Mean wage** | < 1 MW | 3.5 | 5 |
|  | 1 MW | 84.5 | 120 |
|  | >1 to 1.5 MW | 12 | 17 |
| **Time working in school foodservice** | < 1 year | 12 | 17 |
|  | 1-5 years | 38.7 | 55 |
|  | 6-10 years | 16.9 | 24 |
|  | > 10 years | 32.4 | 46 |
| **Weekly workday** | 20-30 hours | 70.4 | 100 |
|  | 40 hours | 29.6 | 42 |
|  |  |  |  |
|  |  |  |  |
|  |  |  |  |
| **Participation in food hygiene training** | Yes | 86.6 | 123 |
|  | No | 13.4 | 19 |
